# Comparison of first-line tuberculosis treatment outcomes between previously treated and new patients: a retrospective study in Machakos subcounty, Kenya

**DOI:** 10.1093/inthealth/ihaa051

**Published:** 2020-08-28

**Authors:** Johannes Ndambuki, Joseph Nzomo, Lucy Muregi, Chris Mutuku, Francis Makokha, Jonathan Nthusi, Clarice Ambale, Lutgarde Lynen, Tom Decroo

**Affiliations:** Department of Health and Emergency Services, Machakos County, Kenya; Department of Health and Emergency Services, Machakos County, Kenya; Department of Health and Emergency Services, Machakos County, Kenya; Department of Health and Emergency Services, Machakos County, Kenya; Directorate of Research and Innovation, Mount Kenya University, Box 342-01000, Thika, Kenya; Department of Health and Emergency Services, Machakos County, Kenya; Department of Health and Emergency Services, Machakos County, Kenya; Institute of Tropical Medicine-Antwerp, Nationalestraat 155-B-2000, Belgium; Institute of Tropical Medicine-Antwerp, Nationalestraat 155-B-2000, Belgium; Research Foundation Flanders, Brussels, 1000 Brussels, Belgium

**Keywords:** drug susceptibility testing, rifampicin-resistant tuberculosis, undetected resistance

## Abstract

**Background:**

Since 2016, patients with rifampicin-susceptible tuberculosis (TB) have been treated with the 6-month first-line regimen, regardless of treatment history. We assessed treatment outcomes of previously treated and new patients in Machakos subcounty, Kenya.

**Methods:**

We performed a retrospective cohort study in patients started on first-line treatment between 2016 and 2017. Firth's logistic regression was used to estimate the effect of previous treatment on having a programmatic adverse outcome (either lost to follow-up, death, failure) and treatment failure vs treatment success (either cure or completion).

**Results:**

Of 1024 new and 79 previously treated patients, 88.1% and 74.7% were treated successfully, 6.5% and 7.6% died, 4.2% and 10.1% were lost to follow-up and 1.2% and 7.6% had treatment failure, respectively. Previous treatment predicted having a programmatic adverse outcome (adjusted odds ratio [aOR] 2.4 [95% confidence interval {CI} 1.4 to 4.2]) and treatment failure (aOR 7.3 [95% CI 2.6 to 20.4]) but not mortality. Similar correlations were found in 334 new and previously treated patients with confirmed baseline rifampicin susceptibility.

**Conclusion:**

Previously treated patients were more at risk of experiencing a poor treatment outcome, mainly lost to follow-up and treatment failure. Adherence support may reduce lost to follow-up. Rifampicin drug susceptibility testing coverage should increase. More robust retreatment regimens may reduce treatment failure.

## Introduction

According to the World Health Organization (WHO), tuberculosis (TB) is the 10th leading cause of death worldwide, having caused an estimated 1.2 million deaths in 2018 among human immunodeficiency virus (HIV)-negative people and an additional 251 000 deaths among people living with HIV (PLHIV). This ranks TB above HIV/acquired immune deficiency syndrome as the leading cause of death from a single infectious agent.^2^ The prevalence of TB in the world stands at 133 per 100 000. In Kenya, the incidence of TB is 292 cases per 100 000 population.^1^

An important pillar of TB control is effective treatment. At present, the WHO recommends universal rifampicin drug susceptibility testing (DST).^2^ If it is not feasible to test all patients with a new TB episode, risk groups such as previously treated patients should be prioritized.^2^ Patients without proof of initial rifampicin resistance should be treated with a 6-month rifampicin treatment regimen, regardless of treatment history.^3^ In Kenya, these guidelines were implemented in 2017. Before 2017, an 8-month streptomycin-containing re-treatment regimen was used, which consisted of an initial phase of rifampicin, isoniazid, ethambutol and pyrazinamide for 3 months, with streptomycin added during the first 2 months, followed by a continuation phase using isoniazid, rifampicin and ethambutol for 5 months. Since 2017, all new and previously treated patients without proof of resistance to rifampicin were treated with the standard 6-month WHO first-line regimen TB drugs, comprising a 2-month intensive phase of isoniazid, rifampicin, ethambutol and pyrazinamide followed by a 4-month continuation using isoniazid and rifampicin.

However, this practice seems to ignore the correlation between previous treatment and adverse outcomes. Espinal et al.^4^ showed that previously treated patients with pan-susceptible TB treated with an 8-month rifampicin regimen strengthened with streptomycin had worse outcomes than new patients with pan-susceptible TB treated with a 6-month rifampicin regimen. Other studies also showed that outcomes in previously treated patients were worse than in new patients, where previously treated patients were treated with the streptomycin-strengthened regimen.^5^^–^^10^ We therefore assessed the outcomes of previously treated and new patients enrolled on the standard 6-month rifampicin regimen between 2016 and 2017 in Machakos subcounty, Kenya, and in patients with rifampicin-susceptible TB using the Xpert *Mycobacterium tuberculosis* (MTB)/resistance to rifampicin (RIF) test.

## Methods

### Study design

This was a retrospective cohort study.

### Setting

The Machakos level 5 hospital is located in Machakos, the headquarters of Machakos County, about 60 km to the east of Nairobi, Kenya. It is the main referral health facility within the county. Machakos County borders Nairobi and Kiambu Counties to the north, Makueni to the east, Kitui to the south and Kajiado to the west. This hospital serves TB patients within Machakos subcounty and serves as an Xpert MTB/RIF testing centre for 30 TB treatment centres in the subcounty.

TB was diagnosed on clinical signs and/or smear microscopy. Since 2012, rapid molecular rifampicin DST (Xpert MTB/RIF) has been used in the diagnosis of rifampicin-resistant TB. All patients diagnosed with TB were eligible for Xpert MTB/RIF testing. Samples were transported by contracted motorcycle riders to the Xpert MTB/RIF testing centre. Test results are relayed via mobile message texts to the requesting clinician and hard copies are delivered back by the riders. In accord with the national TB guidelines, TB type was categorized as either pulmonary (PTB) or extrapulmonary (EPTB). Unless rifampicin resistance was detected, patients were started on the 6-month standardized WHO first-line regimen. They had a scheduled weekly clinic visit during the 2-month intensive phase and clinic visits every 2 weeks during the 4-month continuation phase. Follow- up smears were done during months 2, 5 and 6 month of treatment in the respective TB centres. When resistance to rifampicin was detected, at baseline or when treatment failure was identified, patients were switched to an MDR-TB treatment regimen.

### Study population and period

All previously treated and new TB cases registered to start the standard first-line 6-month rifampicin regimen at any of 30 TB treatment centres of Machakos subcounty between January 2016 and December 2017 were included, regardless of HIV status and age. Patients diagnosed initially rifampicin-resistant TB were excluded, as they were treated with the MDR-TB treatment regimen.

### Data collection

Data were collected from a routinely used electronic database and complemented with data retrieved from paper-based TB registers. Any personal identifying information, such as name, telephone number and residence, were not collected. Data were coded, using a unique numeric identifier. Variables included gender, age, type of TB (PTB, EPTB), HIV status, Xpert MTB/RIF result and WHO treatment outcome (cure, completion, treatment failure, death, lost to follow-up; definitions are shown in Table 1).^11^

**Table 1.** WHO outcome definitions and composite outcomes used in the analysis

Outcome	Definition
Cured	A PTB patient with bacteriologically confirmed TB at the beginning of treatment who was smear or culture negative in the last month of treatment and on at least one previous occasion
Treatment completed	A TB patient who completed treatment without evidence of failure but with no record to show sputum smear or culture results in the last month of treatment and on at least one previous occasion were negative, either because tests were not done or because results are unavailable
Treatment failed	A TB patient whose sputum smear or culture is positive at month 5 or later during treatment
Died	A TB patient who dies for any reason before starting or during the course of treatment
Lost to follow-up	A TB patient who did not start treatment or whose treatment was interrupted for ≥2 consecutive months
Composite outcomes	
Treatment success	Either cured or treatment completed
Programmatically adverse outcome	Either treatment failed, died or lost to follow-up

### Analysis

We used calculated 95% confidence intervals (CIs) around risk differences for the Firth's logistic regression to estimate the effect of previous treatment on different adverse outcomes, adjusted for gender, age, type of TB and HIV status, overall and in patients with confirmed rifampicin-susceptible TB on Xpert MTB/RIF. Gender, age, type of TB and HIV status were included in the regression, as these factors were previously reported to be associated with TB treatment outcomes.^5^^,^^12^^–^^14^ Adverse outcomes were mortality, having a programmatic (either lost to follow-up, death or failure) adverse outcome and treatment failure. For the different regressions we used treatment success (either cure or completion) as a favourable outcome. Missingness was handled using the missing indicator approach.^15^ Data analysis was performed using Stata version 16.0 (StataCorp, College Station, TX, USA).

## Results

A total of 1104 patients started first-line TB treatment between 2016 and 2017, of whom 1024 (92.8%) had been newly diagnosed and 79 (7.2%) had been previously treated for TB. Compared with new patients, previously treated patients were older (median age 37 vs 33; p=0.02), more likely to be male (81.0% vs 68.6%; p=0.02) and less likely to have EPTB (16.5% vs 27.9%; p=0.03) (Table 2). Overall, the majority (74.6% [823/1104]) tested negative for HIV, with a similar proportion among new patients and previously treated patients.

**Table 2.** Characteristics of patients treated with category 1 treatment regimen between 2016 and 2017, by treatment history

Characteristics	New cases, n (%)	Previously treated, n (%)	p-Value^*^
Total	1024	79	
Gender			0.02
Female	322 (31.4)	15 (19.0)	
Male	703 (68.6)	64 (81.0)	
Age group (years)			0.02
<15	43 (4.2)	0 (0)	
15–<30	339 (33.1)	18 (22.8)	
30–<50	458 (44.7)	48 (60.8)	
≥50	185 (18)	13 (16.5)	
Type of TB			0.03
Pulmonary	739 (72.1)	66 (83.5)	
Extrapulmonary	286 (27.9)	13 (16.5)	
HIV status			0.7
Negative	761 (74.2)	62 (78.5)	
Positive	261 (25.5)	17 (21.5)	
Unknown	3 (0.3)	0 (0)	
Xpert MTB/RIF result			<0.001
Negative	36 (3.5)	11 (13.9)	
MTB detected	351 (34.3)	40 (50.6)	
Not done	637 (62.1)	28 (35.4)	

* χ^2^ test.

Among 1024 new and 79 previously treated patients, 34.3% (n=351) and 50.6% (n=40) had rifampicin-susceptible TB on Xpert MTB/RIF before starting treatment, 62.1% (n=637) and 35.4% (n=28) had no Xpert MTB/RIF result and 3.5% (n=36) and 13.9% (n=11) tested negative on Xpert MTB/RIF, respectively.

Among the 1024 newly diagnosed patients, 88.1% (n=902) were successfully treated (either cured or treatment completed), 1.2% (n=12) experienced treatment failure, 4.2% (n=43) were lost to follow-up and 6.5% (n=67) died. In the previously treated group of 79 patients, 74.7% (n=59) were successfully treated, 7.6% (n=6) reported treatment failure, 10.1% (n=8) were lost to follow-up and 7.6% (n=6) died (Table 3).

**Table 3.** Treatment outcomes of patients treated with category 1 treatment regimen between 2016 and 2017, by treatment history

Characteristics	New cases, n (%)	Previously treated patients, n (%)	p-Value^*^
Total	1024	79	
Cured	563 (55.0)	41 (51.9)	<0.001
Treatment completion	339 (33.1)	18 (22.8)	
Treatment failure	12 (1.2)	6 (7.6)	
Death	67 (6.5)	6 (7.6)	
Lost to follow-up	43 (4.2)	8 (10.1)	
Composite outcomes^a^			<0.001
Success	902 (88.1)	59 (74.7)	
Programmatically adverse	122 (11.9)	20 (25.3)	

* χ^2^ test.

^a^Success: either cured or treatment completed; programmatic adverse outcomes: either died, treatment failure or lost to follow-up.

Two patients were diagnosed with rifampicin-resistant TB on Xpert MTB/RIF after treatment failure. One female previously treated patient had been treated for drug-sensitive TB a year before starting her second treatment with the same first-line regimen. The patient died 2 days after the diagnosis of rifampicin-resistant TB while preparing to start MDR-TB treatment. One male patient tested ‘MTB detected, rifampicin resistance not detected’ on Xpert MTB/RIF at baseline. During treatment, smears did not convert and a repeat Xpert MTB/RIF showed rifampicin resistance. He was started on MDR-TB treatment and was cured.

In previously treated patients, programmatic adverse outcomes (difference 13.4% [95% CI 4.2 to 23.7], p<0.001) and treatment failure (difference 7.9% [95% CI 1.7 to 16.4], p<0.001) were more frequent than in new patients (Table 4). Lost to follow-up was also more frequent among previously treated patients (difference 7.4% [95% CI 0.3 to 16.5], p=0.008) than in new patients. Mortality was similar in both groups (difference 2.3% [95% CI −4.9 to 10.9], p=0.5).

**Table 4.** Differences in outcomes of new and previously treated cases among patients treated with category 1 treatment regimen between 2016 and 2017

	New patients			Previously treated patients				
							Difference in	
							adverse outcomes,	
Outcomes	Success, n	Adverse, n	Percentage^a^	Success, n	Adverse, n	Percentage^a^	% (95% CI)	p-Value
Treatment failure, death or LTFU vs success	902	122	11.9	59	20	25.3	13.4 (4.2 to 23.7)	<0.001
Treatment failure vs success	902	12	1.3	59	6	9.2	7.9 (1.7 to 16.4)	<0.001
Death vs success	902	67	6.9	59	6	9.2	2.3 (−4.9 to 10.9)	0.5
LTFU vs success	902	43	4.6	59	8	11.9	7.4 (0.3 to 16.5)	0.008

a Number with adverse outcome divided by the same plus the number with success.

LTFU: lost to follow-up.

Overall (N=1103), previous treatment predicted having a programmatic adverse outcome (adjusted odds ratio [aOR] 2.4 [95% CI 1.4 to 4.2]) and treatment failure (aOR 7.3 [95% CI 2.6 to 20.4]) but not mortality (Table 5). Similarly, in 392 patients with rifampicin-susceptible TB on Xpert MTB/RIF, previous treatment predicted having a programmatic adverse outcome (aOR 2.3 [95% CI 1.05 to 5.0]) and treatment failure (aOR 9.2 [95% CI 2.7 to 32.1]) but not mortality (Table 6).

**Table 5.** Comparison of previously treated and new patients on programmatic and bacteriologic adverse outcomes and mortality in 1103 patients treated with first-line TB treatment regimen between 2016 and 2017

		Programmatic adverse outcome			Treatment failure			Mortality		
Characteristics	Success, n	n (%)^a^	OR (95% CI)	aOR (95% CI)	n (%)^a^	OR (95% CI)	aOR (95% CI)	n (%)^a^	OR (95% CI)	aOR (95% CI)
Total	961	142 (12.9)			18 (1.8)			73 (7.1)		
History										
New case	902	122 (11.9)	1	1	12 (1.3)	1	1	67 (6.9)	1	1
Previously treated	59	20 (25.3)	2.5^***^ (1.5 to 4.3)	2.4** (1.4 to 4.2)	6 (9.2)	7.9*** (3.0 to 21.1)	7.3*** (2.6 to 20.4)	6 (9.2)	1.5 (0.6 to 3.4)	1.4 (0.6 to 3.4)
Gender										
Female	292	45 (13.4)	1	1	7 (2.3)	1	1	26 (8.2)	1	1
Male	669	97 (12.7)	0.9 (0.6 to 1.4)	1 (0.7 to 1.5)	11 (1.6)	0.7 (0.3 to 1.7)	0.5 (0.2 to 1.5)	47 (6.6)	0.8 (0.5 to 1.3)	1 (0.6 to 1.7)
Age group (years)										
<15	41	2 (4.7)	1	1	1 (2.4)	1	1	1 (2.4)	1	1
15–<30	327	29 (8.1)	1.5 (0.4 to 5.7)	1.5 (0.4 to 6.0)	2 (0.6)	0.2 (0.0 to 1.6)	0.1* (0.0 to 0.8)	9 (2.7)	0.8 (0.1 to 4.6)	1.3 (0.2 to 7.6)
30–<50	429	77 (15.2)	3.0 (0.8 to 11.0)	2.5 (0.7 to 9.4)	11 (2.5)	0.7 (0.1 to 4.2)	0.3 (0.1 to 2.0)	39 (8.3)	2.5 (0.5 to 13.4)	3 (0.6 to 16.3)
≥50	164	34 (17.2)	3.5 (0.9 to 13.1)	3.5 (0.9 to 13.2)	4 (2.4)	0.8 (0.1 to 5.0)	0.4 (0.1 to 3.1)	24 (12.)8	4.1 (0.8 to 22.2)	5.8* (1.0 to 31.9)
Type of TB										
Pulmonary	697	107 (13.3)	1	1	18 (2.5)	1	1	42 (5.7)	1	1
Extrapulmonary	264	35 (11.7)	0.9 (0.6 to 1.3)	0.8 (0.6 to 1.3)	0 (0)	0.1 (0.0 to 1.2)	0.1 (0.0 to 1.1)	31 (10.5)	2.0^**^ (1.2 to 3.2)	1.8* (1.1 to 2.9)
HIV status										
Negative	735	87 (10.6)	1	1	13 (1.7)	1	1	36 (4.7)	1	1
Positive	223	55 (19.8)	2.1*** (1.4 to 3.0)	2.1*** (1.4 to 3.1)	5 (2.2)	1.3 (0.5 to 3.7)	1.2 (0.4 to 3.6)	37 (14.2)	3.4*** (2.1 to 5.5)	3.1*** (1.8 to 5.2)
Unknown	3	0 (0)	1.2 (0.1 to 23.4)	1.6 (0.1 to 31.5)	0 (0)	7.8 (0.41 to 58.2)	15.9 (0.64 to 00.9)	0 (0)	2.9 (0.1 to 56.8)	4.4 (0.2 to 90.0)

*P<0.05; **P<0.01; ***P< 0.001.

a Proportion with adverse outcome=number of patients with an adverse outcome divided by the same+number with treatment success. Success: either cured or treatment completed; programmatic adverse outcome: either died, treatment failure or lost to follow-up.

Firth's logistic regression was to reduce bias related to the small number of patients with events. The area under the curve for the multivariable logistic regression models assessing predictors of programmatic adverse outcomes, treatment failure and mortality was 0.66, 0.76 and 0.75, respectively.

**Table 6.** Comparison of previously treated and new patients on programmatic and bacteriologic adverse outcomes and mortality in 392 patients with initially rifampicin-susceptible TB on Xpert MTB/RIF and treated with category 1 treatment regimen between 2016 and 2017

		Programmatic adverse outcome			Treatment failure			Mortality		
Characteristics	Success, n	n (%)^a^	OR (95% CI)	aOR (95% CI)	n (%)^a^	OR (95% CI)	aOR (95% CI)	n (%)^a^	OR (95% CI)	aOR (95% CI)
Total	334	58 (14.8)			11 (3.2)			17 (4.8)		
History										
New case	304	48 (13.6)	1	1	6 (1.9)	1	1	16 (5)	1	1
Previously treated	30	10 (25)	2.2* (1.0 to 4.6)	2.3* (1.05 to 5.0)	5 (14.3)	8.4*** (2.6 to 27.9)	9.2*** (2.7 to 32.1)	1 (3.2)	0.9 (0.2 to 5.0)	0.99 (0.2 to 5.8)
Gender										
Female	91	19 (17.3)	1	1	5 (5.2)	1	1	7 (7.1)	1	1
Male	243	39 (13.8)	0.8 (0.4 to 1.4)	0.9 (0.4 to 1.6)	6 (2.4)	0.4 (0.1 to 1.4)	0.5 (0.1 to 1.7)	10 (4)	0.5 (0.2 to 1.4)	0.7 (0.3 to 2.1)
Age group (years)										
<15	5	1 (16.7)	1	1	1 (16.)7	1	1	0 (0)	1	1
15–<30	113	15 (11.7)	0.5 (0.1 to 3.3)	0.7 (0.1 to 4.6)	2 (1.7)	0.1* (0.0 to 0.7)	0.1* (0.01 to 0.8)	2 (1.7)	0.2 (0.0 to 5.7)	0.5 (0.0 to 13.8)
30–<50	164	34 (17.2)	0.8 (0.1 to 4.9)	0.9 (0.1 to 5.7)	6 (3.5)	0.1 (0.0 to 1.0)	0.1 (0.01 to 1.0)	12 (6.8)	0.8 (0.0 to 16.0)	1.4 (0.1 to 27.9)
≥50	52	8 (13.3)	0.6 (0.1 to 4.2)	0.7 (0.1 to 5.4)	2 (3.7)	0.2 (0.0 to 1.6)	0.2 (0.02 to 1.9)	3 (5.5)	0.7 (0.0 to 16.1)	1.5 (0.1 to 36.2)
HIV status										
Negative	241	34 (12.4)	1	1	7 (2.8)	1	1	5 (2)	1	1
Positive	93	24 (20.5)	1.8* (1.0 to 3.2)	1.8 (0.95 to 3.3)	4 (4.1)	1.5 (0.5 to 5.1)	1.2 (0.3 to 4.7)	12 (11.4)	5.9*** (2.1 to 16.5)	4.6** (1.5 to 13.8)

*P< 0.05; **P< 0.01; ***P< 0.001.

a Proportion with adverse outcome=number of patients with an adverse outcome divided by the same+number with treatment success. Success: either cured or treatment completed; programmatic adverse outcome: either died, treatment failure or lost to follow-up.

Firth's logistic regression was to reduce bias related to the low number of patients with events. The area under the curve for the multivariable logistic regression models assessing predictors of programmatic adverse outcomes, treatment failure and mortality was 0.63, 0.83, and 0.75, respectively.

Gender was not associated with having an adverse outcome. Overall, patients ≥50 y of age and patients with EPTB were at risk of mortality (aOR 5.8 [95% CI 1.0 to 31.9]) but not of other adverse outcomes. Overall, and in patients with rifampicin-susceptible TB on Xpert MTB/RIF, HIV/TB co-infected patients were at risk of having a programmatic adverse outcome and mortality but not of having treatment failure.

## Discussion

In our study, previously treated patients without proof of rifampicin resistance were treated with the same 6-month rifampicin first-line regimen as new patients. We studied the effect of the implementation of the 2017 WHO TB guidelines. This guideline recommends to abandon the streptomycin-strengthened 8-month rifampicin re-treatment regimen and to replace it with the 6-month rifampicin regimen in patients without evidence of initial rifampicin resistance or evidence of initial isoniazid resistance.^3^ Overall, previously treated patients were more at risk of having a treatment failure or being lost to follow-up, but not more at risk of mortality. Remarkably, similar findings were seen in those with TB confirmed to be susceptible to rifampicin, the most potent anti-TB drug.^16^

Overall, the odds of treatment failure were 7 times higher (9.2% vs 1.3%) in previously treated patients and 9 times higher (14.3% vs 1.9%) in patients with rifampicin-susceptible TB on Xpert MTB/RIF. A recently published study showed that undetected initial resistance to rifampicin may explain the higher odds of treatment failure in previously treated patients.^17^ In our setting, as in most high TB burden countries,^18^ routine DST for isoniazid is not easily accessible and thus not done in the vast majority of patients. Therefore undetected initial isoniazid may explain excess treatment failures. Gegia et al.^10^ showed a 4-fold (16% vs 4%) higher frequency of recurrence (either treatment failure or relapse) after enrolment on the 6-month regimen in patients with initially isoniazid-resistant/rifampicin-susceptible TB compared with those susceptible to both isoniazid and rifampicin. The same review showed that the 8-month streptomycin-strengthened re-treatment regimen performed slightly better than the 6-month regimen in patients with isoniazid-resistant TB (11% vs 16% recurrence).^10^

Undetected initial isoniazid resistance is likely not the only factor driving the higher odds of treatment failure. In patients with DST for all first-line drugs and with pan-susceptible TB, Espinal et al.^4^ showed a higher frequency of treatment failure among previously treated patients treated with an 8-month rifampicin regimen (35/359 [10%]) than among new patients treated with a 6-month rifampicin regimen (36/786 [4%]). As all were pan-susceptible on baseline DST, the excess of treatment failure was not explained. We speculate that false rifampicin susceptibility may also contribute to the higher odds of treatment failure, especially if a previous rifampicin regimen was unsuccessful. Rapid molecular tests such as Xpert MTB/RIF do not detect all mutations conferring rifampicin resistance. Mutations outside the Rr determining region are missed systematically.^19,^^20^ Depending on the setting, up to 30% of Rr-TB can be missed.^21^ Also rifampicin heteroresistance (mix of mutant and wild-type populations) can be missed by Xpert MTB/RIF.^22^ Moreover, in patients treated with a rifampicin regimen and with TB resistant to rifampicin, the probability of treatment failure is much higher than when isoniazid resistance is missed.^23^

Compared with new patients, previously treated cases were more at risk of being lost to follow-up (11.9% vs 4.6%). In our study, patients with previously treated TB were not treated with a more toxic regimen nor was treatment duration longer. Hence these factors did not explain why previously treated patients were more likely to be lost to follow-up. A previous study showed that those who were lost to follow-up during a previous episode were most at risk of interrupting treatment.^9^ Psychological distress, lack of social support and stigma may occur more often in patients repeatedly treated for TB and may also explain the higher frequency of lost to follow-up.^24^^,^^25^ If patients frequently interrupt treatment, acquisition of resistance may occur.^26^ Therefore, innovative patient support measures beyond directly observed treatment, such as e-health and m-health, are needed, e.g. adherence monitoring through mobile text messaging or smartphone-enabled video-observed treatment.^27^

Treatment success in new patients was 88.1%, close to the 90% End TB target.^2^ To achieve a similarly high success rate in previously treated patients, we will need to study the effect of some interventions. First, we need to ensure access to baseline rifampicin DST for all previously treated patients. In our study, too many previously treated patients did not have baseline rifampicin DST. To achieve close to 100% coverage in those at risk for initial rifampicin resistance should be the first priority. Moreover, in patients not responding well to treatment, rapid molecular rifampicin DST should be repeated. If rifampicin DST again shows ‘rifampicin resistance not detected’, phenotypic DST should be performed to identify resistance to isoniazid and resistance to rifampicin missed by molecular testing. Once the needs of high-risk groups are covered, the next aim could be universal rifampicin DST, including new patients. Second, a more robust re-treatment regimen needs to be designed. At present, rapid isoniazid DST is not decentralized in most high TB burden countries. Ideally, previously treated patients with rifampicin-susceptible TB would be treated with a regimen that is highly effective in patients with initially isoniazid-resistant TB. An alternative to the currently abandoned streptomycin-strengthened re-treatment, which was designed to overcome eventual isoniazid resistance, may be the use of high-dose first-line regimens. Indeed, high-dose isoniazid may overcome mutations conferring resistance to isoniazid for which a normal dose may not be effective.^28^^–^^30^ Also high-dose rifampicin has been shown to be more effective than the normal dose.^31^ To strengthen re-treatment regimens by using a higher dose of first-line drugs requires further study.^30^

Advanced age and HIV co-infection were identified as predictors of mortality, which is consistent with findings from other studies.^12^^,^^13^ In the elderly, comorbidity and delayed diagnosis may contribute to higher mortality.^32^ In HIV co-infected patients, timing of antiretroviral treatment initiation can reduce mortality.^13^

Our study has several strengths. The findings reflect the reality of our TB programme and may be generalized to other settings where the standard first-line treatment regimen is used repeatedly in re-treatment patients with rifampicin-susceptible TB on Xpert MTB/RIF. The standard of care was uniform and standardized definitions were used for clinical variables across all reporting TB units. Personnel responsible for routine reporting were conversant with these definitions. Missing data in the study database were completed by consulting paper-based source documents. Data on HIV status were missing for 3 patients (0.3% of 1104 patients). Given that the sample was large and that HIV co-infection was not rare (25.2% of 1104), we believe bias due to missingness was minimal. Data cleaning involved comparing electronic and paper-based data sources in case of inconsistencies. The main limitation of our study was the relatively low coverage of rifampicin DST, particularly in new patients. Moreover, we had no data on initial isoniazid resistance, thus could not assess the effect of initial isoniazid resistance on outcomes. Neither could we identify initial rifampicin resistance missed by Xpert MTB/RIF but detected on phenotypic DST. We propose to study the frequency and effect of initial resistance to isoniazid and undetected rifampicin resistance.

## Conclusions

The findings of this study show that TB treatment outcomes in Machakos subcounty were generally good, particularly in new patients, but still below the 90% target. Previously treated TB patients were more at risk of experiencing poor treatment outcomes, mainly lost to follow-up and treatment failure. We recommend to enhance adherence support to reduce lost to follow-up and increase coverage of rifampicin DST at baseline. Targeted rifampicin DST for patients not responding to treatment and phenotypic rifampicin DST in patients at risk of Rr-TB not responding to first-line treatment but rifampicin susceptible on rapid molecular rifampicin DST may identify patients in need of second-line TB treatment. More robust re-treatment regimens may overcome initial resistance to isoniazid and reduce treatment failure.
